# Distribution of Heavy Metals in Surface Sediments of the Bay of Bengal Coast

**DOI:** 10.1155/2017/9235764

**Published:** 2017-01-31

**Authors:** M. Z. H. Khan, M. R. Hasan, M. Khan, S. Aktar, K. Fatema

**Affiliations:** ^1^Department of Chemical Engineering, Jessore University of Science and Technology, Jessore 7408, Bangladesh; ^2^DRiCM, Bangladesh Council of Scientific and Industrial Research, Dhaka, Bangladesh

## Abstract

The concentrations of major (Si, Al, Ca, Fe, and K) and minor (Cd, Mn, Ni, Pb, U, Zn, Co, Cr, As, Cu, Rb, Sr, and Zr,) elements in the surficial sediments were studied in an attempt to establish their concentration in the Bengal coast. It was revealed that the majority of the trace elements have been introduced into the Bengal marine from the riverine inflows that are also affected by the impact of industrial, ship breaking yard, gas production plant, and urban wastes. The concentration of heavy metals was measured using Atomic Absorption Spectroscopy and Energy Dispersive X-ray fluorescence instruments. The highest concentrations for several trace elements were thus recorded which generally decrease with distance from the coast. It was observed that the heavy metal concentrations in the sediments generally met the criteria of international marine sediment quality. However, both the contamination factor and pollution load index values suggested the elevation of some metals' concentrations in the region. Constant monitoring of the Bengal coast water quality needs to be recorded with a view to minimizing the risk of health of the population and the detrimental impacts on the aquatic ecosystem.

## 1. Introduction

In recent years, heavy metals pollution of the aquatic environment has become a worldwide problem. Toxic pollutants, such as heavy metals, originate from direct atmospheric deposition or geologic weathering or through the discharge of industrial waste products deposited in marine sediments as a sink. Due to their potential toxic effect and ability to bioaccumulate in aquatic ecosystems [1, 2], the investigation of distribution and pollution degree of heavy metals in coastal area has attracted more public concerns recently [3–9].

The potential sources of heavy metal pollution in the aquatic environment are industrial wastes and mining [10]. Metals like arsenic, cadmium, chromium, mercury, nickel, and lead are often considered indicators of anthropogenic influence in marine environment and are themselves of potential risk to the natural environment [11, 12]. Several researchers have demonstrated that the evaluation of metal distribution in marine surface sediment is important due to high pollution with heavy metals [12, 13]. Therefore, to assess and track the abundance of these heavy metals in coastal ecosystem is an important task [14].

Southern part of Bangladesh is situated at the coast of Bay of Bengal. World's greatest mangrove forest is situated at this part. Chittagong is the biggest port city and also the coastal city of the country, where the pollution problem is acute due to the stress caused by industrial and domestic effluent. Key sources of pollution are mainly from gas production plants, ship breaking yard, and untreated wastes from port, metropolitan, and nearby industries. The nearby industries are discharging waste water directly to the sea without any treatment which is causing serious damage to marine ecology and aquatic lives along with the health of coastal people who are exposed to this environment for a long term. Their effect is more evident from the abnormal values of a set of physical and chemical parameters. The significance of trace elements in marine sediment is increasingly becoming an issue of global concern and needs proper assessment [15, 16]. Thereby, assessment of heavy metals pollution at the Bengal marine coastal area is of considerable interest of both scientific and regulatory communities. So far, there are limited works or no work focusing on heavy metals investigation near this coastal area.

In this study, several sediment samples were collected from Bengal marine bay near Chittagong city of Bangladesh during winter of 2014. It was aimed to determine the heavy metals concentrations and distribution in the sediments from the study area. The main objectives of this work were to reveal the spatial distribution of major and minor elements in the study area and to evaluate the metal concentration using contamination factor (CF) and pollution load index (PLI).

## 2. Materials and Methods

### 2.1. Description of Study Area

**Figure 1:**
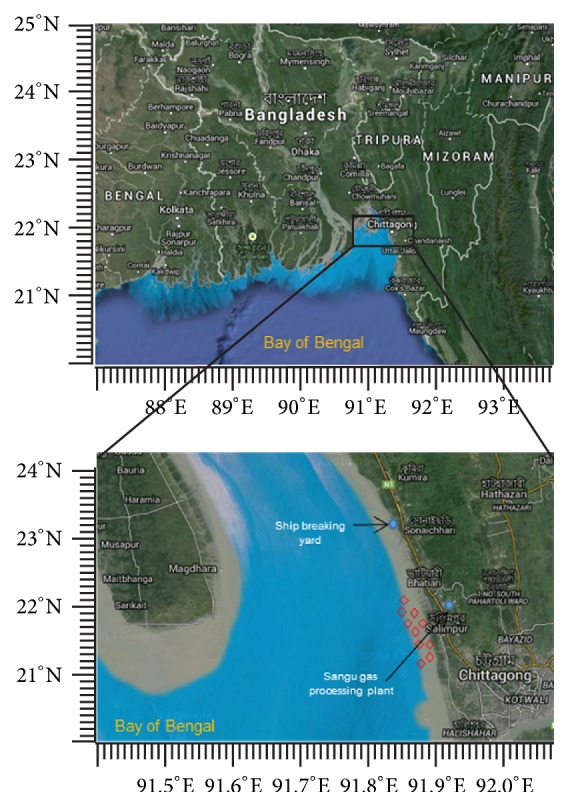
Map of sampling sites along the coast of Bay of Bengal. The image was taken and modified from Google Map, copyright © Google 2015.

For surface sediments sampling, ten sampling points were chosen at the coast of marine bay (Figure 1). The sampling points were located at Salimpur Union, just 10 km from Chittagong city which is the second largest city of Bangladesh. These sites were chosen, because they receive considerable amounts of waste water from industrial areas as well as from ship breaking yard and Sangu gas production plant.

### 2.2. Surface Sediment Sampling

Ten surface sediment samples were collected from Bengal coastal area. Antirust scoop was used to collect the sediment samples by scooping up 10 cm of the bed sediment from 10 m away from the coastal bank and sediments were naturally dried at room temperature (25°C ± 2) in the laboratory prior to analysis. The sampling bottles were preconditioned with 5% nitric acid and later rinsed thoroughly with distilled deionized water. Before sampling was done, the polyethylene sampling bottles were rinsed at least three times. Sediment samples were collected using grab sampler from two sites. Samples were transported to the laboratory and air-dried once; sediment samples were powdered and passed through 160 *µ*m sieve. After packing in polyethylene bags the samples were stored below −20°C prior to analysis. Sediments samples were weighed and placed into the digestion bombs with 10 mL of HNO_3_/HCl (1 : 3 v/v) and digested in a microwave digestion system. Sediments analysis was carried out according to the standard procedure described earlier [3–7].

### 2.3. Instrumentation

The elements determinations were performed by means of a SHIMADZU AAS-7000 (Flame Atomic Absorption spectrometer) for Al, Fe, Ba, Mn, Cu, Ni, Zn, V, Cu, and Pb. The Thermo Scientific ARL QUANT'X Energy Dispersive X-ray fluorescence (EDXRF) spectrometer analyzer was used for determining m/m% for wide band metals. Background corrections were applied whenever required during the analysis and the method of standard additions was used to compensate for matrix effects.

### 2.4. Analytical Quality Control Procedures

Prior to elements analyses, about 20 g of the subsamples were oven-dried at 45°C for 24 h and then ground to pass a 0.125 mm sieve and stored in clean plastic bags at room temperature till measurements were done. Performance of the instrument was checked by analyzing the standard reference material solutions (Fluka, USA) concurrently to check the precision of the instrument. After appropriate dilutions of stock standard solutions, a five-level calibration curve was prepared. Samples were analyzed in triplicate. The values obtained from the sample were corrected for final digestion volume and sample weight was taken. The results were reported on dry weight basis. Duplicate method blanks were also processed and analyzed alongside the samples to check any loss or cross contamination. The differences of the concentrations between the determined and certified values were less than 5%, and the analytical precision for replicate samples was within ±10%. Blanks and Standard Reference Materials were included in the analyses as part of the quality assurance and quality control (QA/QC).

### 2.5. Statistical Analysis

#### 2.5.1. Contamination Factor (CF)

Contamination factor (CF) is usually used to express the level of contamination [17, 18]. The value was calculated as follows:

(1) $$\begin{array}{c} CF=\frac{C_{sample}}{C_{background}}, \end{array}$$

where *C*_sample_ is mean metal content in sample sediment; *C*_background_ is mean natural background value of that metal. Natural background sample was collected from Patenga sea beach area, which is about 20 km from the polluted area.

The PLI is determined as the *n*th root of *n* contamination factors (CF*n*) multiplied together and calculated using the following equation:

(2) $$\begin{array}{c} PLI={\left(\;CF1\times CF2\times CF3\times \cdots \times CFn\;\right)}^{1/n}. \end{array}$$

## 3. Results and Discussion

### 3.1. Determination of Heavy Metals in Sediments

#### 3.1.1. Major Elements

The concentrations of Al and K in the surficial sediments range from 7.28 to 11.65% and from 6.31 to 7.99%, respectively (Table 1). Those elements are primarily a function of clay mineral content and comprise large portion of the surficial sediments [19] and of feldspar, amphiboles, and pyroxenes. The sediments from sample 5 and sample 8 contain the highest concentrations of Al (11.50 ± 0.9%) and K (7.50 ± 0.45%), whereas the lowest concentrations of Al (7.28 ± 0.2%) and K (6.31 ± 0.3%) were observed at sample 4. The sediment which mainly originated from nearby rivers carrying suspended solids which are transported by the marine current along the Bengal coast can be correlated with the geographic distribution pattern of Al and K.

The concentrations of Fe range from 8.95 to 14.56%. Clay minerals are principally associated with the silt-clay fraction; thus they carry more iron than sand grains. In the toxic environment an important role of scavenging heavy metals seems to be played by the Fe oxides/hydroxides [20]. The very high concentration of Fe could be attributed to the effluent from ship breaking yard.

The concentrations of K and Ca range from 6.31 to 7.99 and from 1.99 to 2.93%, respectively. The distribution of concentration is almost the same for all measured samples. Relatively high levels of K (about 7%) and low concentrations of Ca (about 2%) in all samples along the Bengal coast can be attributed to the weathering of basic and ultrabasic igneous rock in the drainage area of the rivers.

Superimposed on these regional patterns are several anomalous elemental concentrations. Iron, potassium, and aluminium are enriched in the sediments on the upper slope compared to the down slope. The concentration patterns of iron, potassium, and aluminum in cores from these areas indicate that both elements have become concentrated at the surface, possibly the result of upward migration of these elements within the sediment column. It can be pointed out that the chemical complexes of heavy metals are free to migrate and also act as chemical “sinks” for other elements. This may account for the high concentration of other elements in this region.

#### 3.1.2. Minor Elements

**Figure 2:**
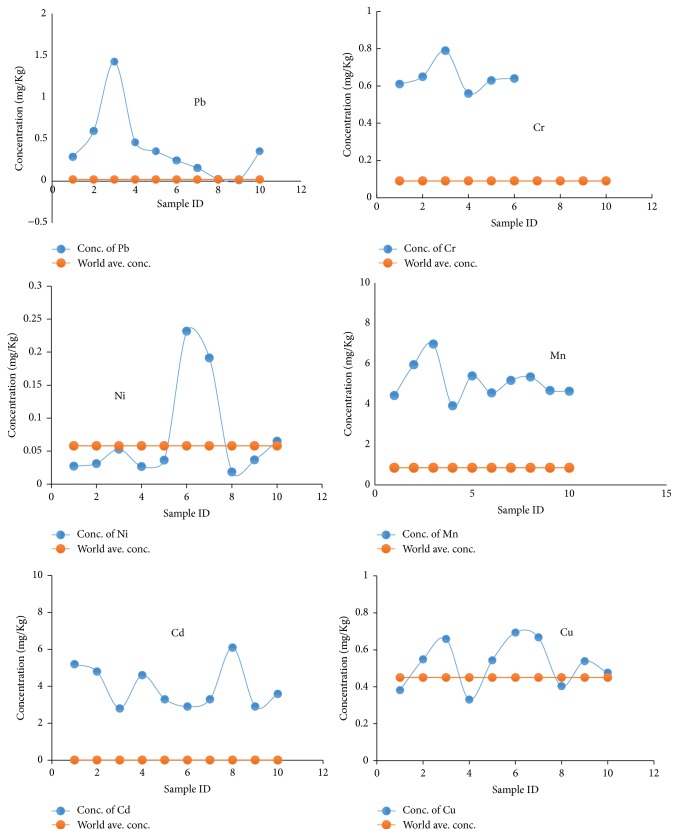
Concentration of minor elements measured versus world average concentration for different surface sediments samples.

Heavy metals released to marine environments act as an ultimate sink into the aquatic environments [21, 22]. This study measured selected heavy metals in sediments for assessment and monitoring of industrial pollution and environmental disasters in the Bengal coast. Total concentrations of heavy metals were analyzed using atomic adsorption spectrometer and ED-XRF analyzer. Standard metal solutions were used for quality control and duplicate analysis. The accumulation of heavy metals Pb, Mn, Ni, Cu, Cd, and Cr was presented in Figure 2, where each element's concentration was compared with world average value of that metal.

The range and average concentrations (mg/kg) were 3.91–6.97 (5.16 ± 1.5) for Mn, 0.01–1.42 (0.4 ± 0.163) for Pb, 0.01–0.23 (0.08 ± 0.001) for Ni, 0.38–0.66 (0.53 ± 0.02) for Cu, 2.8–6.1 (4.0 ± 1.65) for Cd, and 0.61–0.79 (0.74 ± 0.05) for Cr. The high values of Cd were found at all samples, whereas highest levels of Mn (6.97 mg/kg), Pb (1.42 mg/kg), and Cr (0.74 mg/kg) concentrations were observed at sample 3. Cadmium, chromium, and manganese average levels exceeded world average concentration levels for surface sediment [23].

A moderate concentration distribution pattern was observed for Ni in the surface sediments. The reason might be its incorporation in the dispersed skeletal fragments. In the study area, nickel values range from 0.01 to 0.23 mg/kg. The finding of such concentration of Ni in the present study is still rich enough and suggests anthropogenic contribution. Considering the basis of essentiality and over supply, chromium is thought to be one of the least toxic of the minor elements. In ultramafic rocks, Mg and Ni are its lithophile associations that also present in mafic minerals. The ability to form independent Cr minerals makes it more complicated distribution among rock forming minerals [24]. In the study area, chromium values range from 0.61 to 0.79 mg/kg. Almost same concentration was observed for all study samples.

The highest values of Pb (1.42 mg/kg) and Mn (6.97 mg/kg) were recorded in sample 3; those were much higher than the standard value. This may be attributed to the various sources, such as industrial effluents, waste water from gas production plant and ship breaking yard, sewage outfall from port city, and other local factories. The main sources of these elements are thought from anthropogenic input, such as contribution of leaded fuel from automobiles and car batteries and extensive use of the antifouling paints by shipping activities.

Cadmium is highly toxic to most plants and animal species, of which the main anthropogenic sources relate to metallurgical industries, mine wastes, sewage sludge, and municipal effluents. A high level of Cd was detected in all samples (average: 4.0 ± 1.65). Several factors including municipal runoff, atmospheric deposition, and domestic and industrial effluents are responsible for the high levels of Cd and Cu content. It was reported by many researchers that the effluents from the urban areas are the main cause for the elevated levels of trace metals in the aquatic systems.

Table 2 shows the concentration of some trace elements in the measured sediment samples. The average concentrations (mg/kg) were 2.97 ± 0.7 for Ba, 1.92 ± 0.23 for U, 2.57 ± 0.16 for Zr, 0.74 ± 0.3 for Rb, and 0.59 ± 0.13 for Sr. From the measured value, it was observed that the trace metals concentration is within normal range limit; thereby no contamination level was detected. Metal concentrations variations in the sediments of accumulation zones and transportation zones are apparently lower. The degrees of pollution do not necessarily vary with differing sediment trace metal contents.

### 3.2. Evaluation of Sediment Pollution

The overall metal contamination assessment is a difficult task due to the unknown natural background in the sediments. In this study, two widely accepted approaches were employed to evaluate the sediment pollution: contamination factor (CF) and pollution load index (PLI) [25–29]. The control samples were taken to represent natural background metal concentration value. PLI is able to give an estimate of the metal contamination obtained as a contamination factor (CF) of each metal with respect to the natural background value [30, 31].

There are mainly four classifications to express contamination factor: CF < 1 refers to the low contamination factor, 1 ≤ CF < 3 refers to the moderate contamination factor, 3 ≤ CF < 6 refers to the considerable contamination factor, and CF ≥ 6 refers to the very high contamination factor. The values of CF factor are shown in Table 3. In general, the increasing order of CF is Ni > Fe > Cd > Mn > Cu > Pb. Very high contamination was recorded at all samples for Ni (30.22 ± 3.19) and Fe (17.80 ± 3.28), whereas considerable contamination was observed for Mn (3.43 ± 0.35) and Cd (4.07 ± 0.54). Low level contamination factor was found for Cu (3.03 ± 0.45) and Pb (2.42 ± 0.5) at all samples, except at sample 3, which revealed very high contamination factor for Pb (8.70).

#### 3.2.1. Pollution Load Index (PLI)

**Figure 3:**
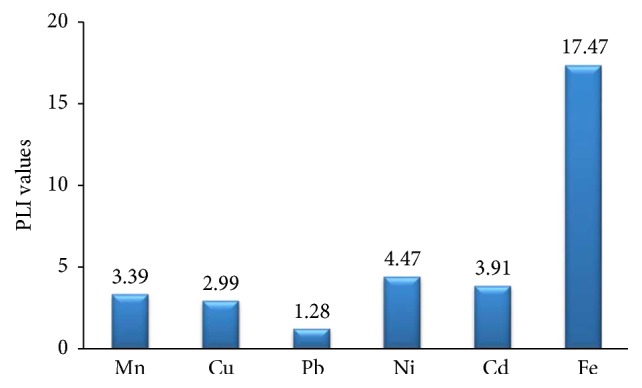
Pollution load index (PLI) values for heavy metals in sediments.

To effectively decide whether the sampling sites suffer contamination or not, the pollution load index (PLI) was used. PLI values of the analyzed samples are shown in Figure 3. This empirical index provides a simple, comparative means for assessing the level of heavy metal pollution. PLI value > 1 indicates a polluted condition, while PLI < 1 means no metal pollution existing (Tomlinson et al., 1980). PLI values in liquid samples for all metals are much bigger than 1 and hence metal pollution exists in the experimental sites. PLI values observed were > 1 for all metals, which means all samples are metal-contaminated to some extent. The results of average CFs are as high as 17.47 for Fe and as low as 1.28 for Pb. PLI values in sediment samples for all metals are also found to be bigger than 1 as shown in Figure 3 and hence metal pollution exists in the experimental sites.

The high concentrations of Fe can be found in the sediments from industry effluents and drilling operations during gas production. The value of Cd is consistently higher than Pb in the sediment samples which is associated with the carbonate fraction and concentrates on the suspended matter. On the other hand, Pb was mainly associated with the Fe-Mn oxide fraction and had high retention in sediment. The observed high level of Pd, Zn, Co, Cu, and Cr is due to the domestic and industrial effluents and mainly precipitated as soluble oxide. A residual fraction of Cr is buried in the bottom sediments as insoluble compounds.

Generally, the element mobilization in the sediment environment is dependent on physiochemical changes in the water at the sediment-water interface. The precipitation of heavy metal elements in the form of insoluble hydroxides, oxides, and carbonates might be the result of alkaline pH. The minor elements such as Cr, Cu, and Co have interacted with organic matter in the aqueous phase and settled, resulting in a high concentration of these elements in the sediment.

## 4. Conclusion

In recent years, the impact of the wastes discharged in Bengal Sea has been significant due to the high effluent discharge from ship breaking yard and gas production plant effluents. It was observed that the heavy metal concentrations in the Bengal coast sediments were remarkably high and varied among sampling points. For evaluating sediment metal contamination, the combined use of different approaches facilitates a comprehensive interpretation of the sediment characteristics in terms of the background influences. The data analyses by CF factor and PLI value indicated contamination by Ni, Fe, and Cd that values exceeded the limitation of world average concentration. The results suggest that special attention must be given to the issue of element remobilization, because a large portion of elements in sediments are likely to release back into the water column. Therefore, constant monitoring of the water quality is needed to record any alternation in the quality and mitigate outbreak of health disorders and the detrimental impacts on the aquatic ecosystem.

## Figures and Tables

**Table 1:** Measured concentrations of major elements in the coastal sediments.

	Si (%)	Fe (%)	Al (%)	K (%)	Ca (%)
Sample 1	63.81	12.97	10.34	7.33	2.55
Sample 2	63.44	13.6	10.34	7.79	2.9
Sample 3	71.96	8.95	7.83	6.63	2.93
Sample 4	73.36	9.02	7.28	6.31	1.99
Sample 5	62.91	13.4	11.65	7.6	2.4
Sample 6	66.93	10.92	10.6	7.28	2.39
Sample 7	62.77	14.56	10.11	7.99	2.28
Sample 8	63.09	13.48	11.47	7.76	2.08

**Table 2:** Measured concentrations of trace elements in the coastal sediments (mg/kg).

	Ba	U	Zr	Rb	Sr
Sample 1	3.13	2.15	1.42	1.33	0.64
Sample 2	2.70	1.45	1.01	0.71	0.63
Sample 3	4.54	3.64	1.65	1.19	0.82
Sample 4	2.32	2.98	0.95	0.04	0.63
Sample 5	1.80	0.65	1.36	0.57	0.03
Sample 6	2.87	1.08	1.70	0.60	0.57
Sample 7	3.56	1.98	3.01	0.89	0.81
Sample 8	2.86	1.48	1.44	0.70	0.65

**Table 3:** Contamination factor (CF) calculation for minor elements in sediment samples.

Element	CF factor									Natural background concentration^a^ (mg/Kg)
	S-1	S-2	S-3	S-4	S-5	S-6	S-7	S-8	S-9	
Mn	2.9	3.9	4.6	2.6	3.6	3.0	3.4	3.5	3.1	1.5
Pb	1.7	3.6	8.7	2.8	2.1	1.5	0.9	0.1	0.1	0.163
Fe	16.4	17.6	21.6	11.8	17.2	14.6	24.4	18.0	18.5	16.2
Ni	27.1	31.2	53.0	26.7	36.4	23.2	19.3	18.3	36.8	0.001
Cu	2.2	3.1	3.8	1.9	3.1	4.0	3.8	2.3	3.1	0.0274
Cd	5.3	4.9	2.8	4.7	3.3	2.9	3.3	6.2	2.9	1.65

^a^Average natural background concentration (*n* = 3) obtained in the study; S: sample.
